# High level of viral microRNA-BART20-5p expression is associated with worse survival of patients with Epstein-Barr virus-associated gastric cancer

**DOI:** 10.18632/oncotarget.14744

**Published:** 2017-01-19

**Authors:** Byung Woog Kang, YongHun Choi, Oh Kyoung Kwon, Seung Soo Lee, Ho Young Chung, Wansik Yu, Han Ik Bae, An Na Seo, Hyojeung Kang, Suk Kyeong Lee, Seong Woo Jeon, Keun Hur, Jong Gwang Kim

**Affiliations:** ^1^ Department of Oncology/Hematology, Kyungpook National University Hospital, Kyungpook National University School of Medicine, Kyungpook National University Cancer Research Institute, Kyungpook National University, Daegu, South Korea; ^2^ Department of Biochemistry and Cell Biology, Cell and Matrix Research Institute, School of Medicine, Kyungpook National University, Daegu, South Korea; ^3^ BK21 Plus KNU Biomedical Convergence Program, Department of Biomedical Science, Kyungpook National University, Daegu, South Korea; ^4^ Department of Surgery, Kyungpook National University Hospital, Kyungpook National University School of Medicine, Daegu, South Korea; ^5^ Department of Pathology, Kyungpook National University Hospital, Kyungpook National University School of Medicine, Daegu, South Korea; ^6^ College of Pharmacy, Institute of Microorganisms and Research Institute of Pharmaceutical Sciences, Kyunpook National University, Daegu, South Korea; ^7^ Department of Medical Lifescience, College of Medicine, Catholic University of Korea, Seoul, South Korea; ^8^ Department of Gastroeneterology, Kyungpook National University Hospital, Kyungpook National University School of Medicine, Daegu, South Korea

**Keywords:** gastric cancer, epstein-barr virus, microRNA, prognosis, BART

## Abstract

This study analyzed the relationship between several Epstein-Barr virus (EBV) microRNA (miRNA) expression profiles and the clinicopathologic features of patients with EBV-associated gastric cancer. The miRNA expression was examined in 59 tumor and 39 paired normal mucosal tissues from available formalin-fixed paraffin embedded tissue samples. The expression levels of miR-BamHI fragment A rightward transcript (BART)1-5p, miR-BART4-5p, and miR-BART20-5p were determined using a quantitative real-time polymerase chain reaction. The expression of all three analyzed EBV microRNAs was significantly higher in the tumor tissue than in the paired normal tissue (*P* < 0.001 for each). When the median value of the EBV microRNA expression levels was used as the cutoff point, a high BART20-5p expression was associated with worse recurrence-free survival (*P* = 0.034) in a multivariate analysis including age and pathologic stage. In conclusion, the expression level of BART20-5p may predict recurrence-free survival for patients with EBV-associated gastric cancer. Further studies are warranted to clarify the roles of EBV BART microRNAs in the carcinogenesis, and their potential as a biomarker and therapeutic target for EBV-associated gastric cancer.

## INTRODUCTION

Based on a comprehensive molecular characterization of gastric cancer, Epstein-Barr virus (EBV)-associated gastric cancer (EBVaGC) has been identified as one of four gastric cancer subtypes and includes the following distinct characteristics: CDKN2A promoter hypermethylation, recurrent PIK3CA mutations, PD-L1/2 overexpression, and sometimes immune cell signaling activation [1]. These differentiated features are associated with the expression of many viral gene products by EBV infection [2]. EBVaGC belongs to latency type I where the viral genes EBV nuclear antigen 1 (EBNA1), EBV-encoded small RNA (EBER1/2), BamHI-A right-ward transcripts (BARTs), and latent membrane protein 2A (LMP2A) may be expressed [3]. In particular, EBV-encoded microRNAs (miRNAs) target viral genes to mediate immune evasion or maintenance of latency, and incorporate into RNA-induced silencing complex can also interact with specific host genes directly or indirectly [4].

MiRNAs are a class of short, single-strand noncoding RNAs that regulate gene expression by translational repression or mRNA degradation of the target [5, 6]. EBV miRNAs can be subdivided into two groups, BART miRNAs and Bam HI fragment H rightward open reading frame 1 (BHRF1) miRNAs, based on their locations [4]. To date, 22 pre-miRNAs and 40 matures miRNAs have been identified originating from the BamHI-A region [7]. Previous reports have shown high-level expression of EBV BART miRNAs in EBV-associated epithelial cancers, thereby inhibiting the expression of different genes [8–11]. For instance, miR-BART4-5p suppressed proapoptotic protein Bid (BH3-interacting domain death agonist) expression, leading to reduced apoptosis [12]. Recent study also found that miR-BART20-5p reduced BAD (Bcl-2-associated death promoter) expression and inhibition of BAD by using a miR-BART20-5p mimic promoted cell proliferation [13, 14]. Kanda *et al*. reported a relationship between BART miRNA expression and epithelial carcinogenesis *in vivo*. In particular, it was shown that N-myc downstream regulated gene 1 (NDRG1) protein, which is a putative target of BART miRNAs, can be used as an epithelial differentiation marker and a suppressor of metastasis [15]. However, the clinical outcomes of most BART miRNAs remain unclear in patients with EBVaGC. Since viral miRNAs may be useful as biomarkers for EBVaGC, this study analyzed the relationship between several EBV miRNA expressions and the clinicopathologic features of patients with EBVaGC.

## RESULTS

### Patient characteristics

The baseline characteristics of the patients are described in Table 1. The median age was 64 years (range, 40–78) and 79.7% (47/59) were male. The majority of the tumors (57.6%) were located in the body of the stomach, and the pathologic TNM stage was I, II, and III in 20 (33.9%), 21 (35.6%), and 18 (30.5%) patients, respectively. Twenty-two patients were classified as gastric carcinoma with lymphoid stroma (GCLS). The median value of the EBV miRNA expression levels was used as the cutoff point. Among the 59 patients, 29 and 28 were determined as the BART1-5p-high and BART20-5p-high expression group, respectively. Plus, 20 out of 39 cases were BART4-5p-high expression.

**Table 1:** Baseline patient characteristics of tumor tissues

Characteristic	Total (*N* = 59) *N* (%)
Age, years	
Median (Range)	65.0 (40.0–78.0)
Gender	
Male	47 (79.7)
Female	12 (20.3)
Tumor location	
Gastroesophageal junction, cardia and fundus	6 (10.2)
Body	34 (57.6)
Antrum	6 (10.2)
Others	13 (22.0)
Treatment	
Surgical resection	59 (100.0)
Tumor depth (pathologic T category)	
T1	16 (27.1)
T2	7 (11.9)
T3	18 (30.5)
T4	18 (30.5)
Lymph node metastasis (pathologic N category)	
N0	31 (52.5)
N1	10 (16.9)
N2	7 (11.9)
N3	11 (18.6)
pTNM stage	
I	20 (33.9)
II	21 (35.6)
III	18 (30.5)
Lauren classification	
Intestinal	7 (11.9)
Diffuse	47 (79.7)
Mixed	5 (8.5)
WHO classification	
Gastric carcinoma with lymphoid stroma (GCLS)	22 (37.3)
Non-GCLS	37 (62.7)

pTNM, pathologic tumor-node-metastasis.

### BART miRNA expression in tumor tissues and association with pathologic features

**Figure 1:**
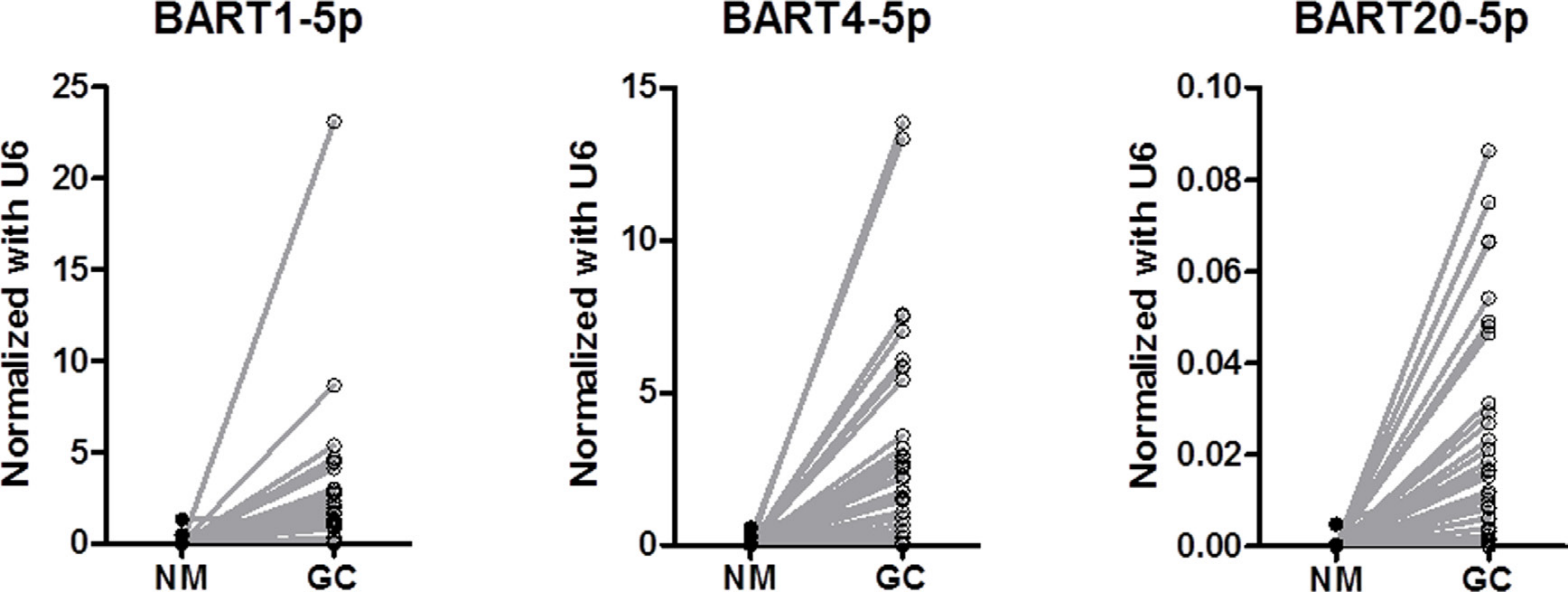
Comparison of expression levels of BART miRNAs between tumor tissue and paired normal tissue (*P* < 0.001 for each)

Figure 1 shows the EBV BARTs miRNA expression in the stomach tumor tissue and paired normal tissue, where miRNA expression of BART1-5p, BART4-5p, and BART20-5p was readily detected in all the EBVaGC cases. Plus, as expected, the expression of these EBV BART miRNAs was significantly higher in the tumor tissues than in the paired normal tissues (*P* < 0.001 for each). The relationship between the BART miRNA expression and the clinicopathologic features was also analyzed (Table 2). As a result, the clinicopathologic features were similar between the two groups according to the expression of BARTs miRNA.

**Table 2:** Association between clinicopathologic features and BART miRNAs expression

	miR-BART1-5p (N = 58)			miR-BART4-5p (N = 39)			miR-BART20-5p (N = 57)		
	Low	High	P	Low	High	P	Low	High	P
Gender			0.747			0.451			0.735
Male	22 (75.9)	24 (82.8)		14 (73.7)	17 (85.0)		30 (78.9)	16 (84.2)	
Female	7 (24.1)	5 (17.2)		5 (26.3)	3 (15.0)		8 (21.1)	3 (15.8)	
Age			0.058			0.882			0.336
< 64	7 (24.1)	15 (51.7)		9 (47.4)	9 (45.0)		13 (34.2)	9 (84.2)	
≥ 64	22 (75.9)	14 (48.3)		10 (52.6)	11 (55.0)		25 (65.8)	10 (15.8)	
T stage			0.788			0.265			0.159
T1/2	12 (41.4)	11 (37.9)		9 (47.4)	6 (30.0)		18 (47.4)	5 (26.3)	
T3/4	17 (58.6)	18 (62.1)		10 (52.6)	14 (70.0)		20 (52.6)	14 (73.7)	
LN metastasis			0.293			0.429			0.260
No	17 (58.6)	13 (44.8)		10 (52.6)	8 (40.0)		22 (57.9)	7 (42.1)	
Yes	12 (41.4)	16 (55.2)		9 (47.4)	12 (60.0)		16 (42.1)	11 (57.9)	
WHO classification			0.588			0.651			0.847
GCLS	12 (41.4)	10 (34.5)		7 (36.8)	6 (30.0)		15 (39.5)	7 (36.8)	
Non-GCLS	17 (58.6)	19 (65.5)		12 (63.2)	14 (70.0)		23 (60.5)	12 (63.2)	
Lauren classification			0.456			0.301			0.430
Intestinal	5 (17.2)	2 (6.9)		1 (5.3)	2 (10.0)		6 (15.8)	1 (5.3)	
Diffuse	22 (75.9)	24 (82.8)		14 (73.7)	17 (85.0)		30 (78.9)	16 (84.2)	
Mixed	2 (6.9)	3 (10.3)		4 (21.1)	1 (5.0)		2 (5.3)	2 (10.5)	

LN, lymph node metastasis; GCLC, Gastric carcinoma with lymphoid stroma.

### Effect of BART miRNA expression on survival

**Figure 2:**
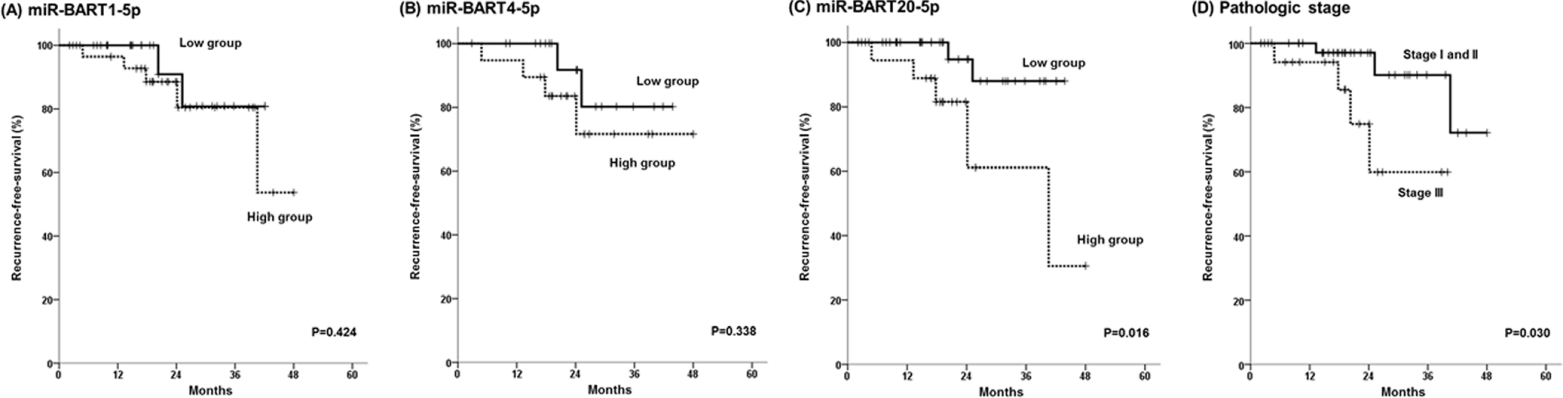
Kaplan-Meier survival curves for recurrence-free survival according to (**A**) expression levels of miR-BART1-5p, (**B**) miR-BART4-5p, (**C**) miR-BART20-5p, and (**D**) pathologic stage.

With a median follow-up duration of 24.1 months (2.8–48.0), the estimated 3-year recurrence-free survival (RFS) and overall survival (OS) rates for all 59 patients were 76.4% and 78.7%, respectively. In a multivariate analysis adjusted for age and stage, a high expression of miR-BART20-5p showed a statistically significant correlation with shorter RFS (*P* = 0.034, HR = 6.951, 95% CI = 1.158–41.737)(Figure 2 and Table 3). Meanwhile, miR-BART1-5p and miR-BART4-5p expression was not found to be associated with RFS in the survival analysis (Figure 2).

**Table 3:** Survival analysis for recurrence-free survival

Variables	Category	Recurrence-free survival in all patients			
		Univariate analysis		Multivariate analysis	
		P	P	HR	95% CI
Age, years	< 64 vs ≥ 64	0.666	0.097	5.209	0.743–36.514
pTNM Stage	I & II vs. III	0.030	0.050	6.630	0.999–44.012
miR-BART20-5p	High vs. low	0.016	0.034	6.951	1.158–41.737

pTNM, pathologic tumor-node-metastasis.

## DISCUSSION

The prognostic impact of BART miRNAs was investigated in EBVaGC patients who had been curatively resected. As a result, the current study demonstrated that a high-level expression of miR-BART20-5p was associated with poor survival for the EBVaGC patients. This study also seem to be noteworthy that EBV BART miRNAs were readily detectable in EBVaGC tumor tissues in contrast to the paired normal tissues.

To date, > 40 mature EBV BART miRNAs have been identified and these miRNAs are derived from BART introns prior to splicing [15, 16]. Although the functions of most BART miRNAs remain unknown, BART miRNAs are expressed at high levels in EBV-associated epithelial malignancies, including EBVaGC. Consistent with these results, the current study also found readily detectable levels of BART miRNA expression in the EBVaGC tissues, suggesting that BART miRNAs may contribute to epithelial tumorigenesis. Several studies have already investigated the functional effect of various BART miRNAs on target genes. Choi *et al*. reported that miR-BART15 is preferentially secreted from EBV-infected gastric carcinoma cells via exosome and can increase cell apoptosis [17]. Plus, other studies found that miR-BART5-5p targets a p53-upregulated modulator of apoptosis to promote cell survival [18], while a translocase of the outer mitochondrial membrane 22 homolog is a potential target of miR-BART16 [19]. More recently, Shinozaki-Ushiku *et al*. reported that reduction of apoptosis in clinical samples from EBVaGC patients was attributable to the expression of miR-BART4-5p which suppress proapoptotic protein Bid [12]. These results suggest that BART miRNAs may be involved in activation of several key cancer-related proteins.

In the present study, the patients with low miR-BART20-5p expression showed a better RFS compared to the patients with high miR-BART20-5p expression, which is consistent with our previous report, where miR-BART20-5p reduced BAD expression in EBV-infected gastric cancer cells by directly targeting the 3*′*-untranslated region of BAD [13]. miR-BART20-5p also suppressed lytic induction both directly [20] and indirectly by inhibiting BAD-mediated caspase-3-dependent apoptosis [1, 14]. Thus, given the function of miR-BART20-5p, the present results, which showed an inverse relationship between survival and the expression level of this viral miRNA, suggest that miR-BART20-5p may act as a therapeutic target in EBVaGC.

Nonetheless, despite the higher expression of miR-BART1-5p and miR-BART4-5p, their prognostic significance was not clearly elucidated. Thus, the carcinogenic roles of EBV miRNAs need to be further identified in order to determine their prognostic relevance. According to a recent miRNA profiling study, the oncogenetic value of BART1-5p was investigated using nasopharyngeal cancer and lymphoblastoid cell lines. As a result, miRNA-BART1-5p was found to suppress the target gene in EBV and also repressed the host cellular protein [10, 13]. Plus, the inhibition of BART4-5p expression has been associated with partial recovery of the apoptotic activator molecule in EBVaGC [15]. It can be speculated that alterations of BART miRNAs may be involved in different mechanisms in various cancer types.

In conclusion, miR-BART1-5p, miR-BART4-5p, and miR-BART20-5p were readily detectable in EBVaGC tissues, and the expression level of BART20-5p may predict RFS for patients with EBVaGC. Further studies are warranted to clarify the roles of EBV BART miRNAs in the carcinogenesis of EBVaGC and their potential as a therapeutic target.

## MATERIALS AND METHODS

### Patients

All the tissue samples used in this study were obtained from patients included in a previous study of EBVaGC, the inclusion criteria and results for which have already been reported [21]. In brief, the study included patients with histologically confirmed adenocarcinoma of the stomach who underwent surgical resection or endoscopic mucosal dissection at Kyungpook National University Medical Center (KNUMC) between January 2011 and November 2014. After reviewing 1318 consecutive cases, 120 patients were identified as EBV-positive using EBV-encoded RNA *in situ* hybridization. Among these 120 patients, miRNA expression was examined in 59 tumor and 39 paired normal mucosal tissues from available formalin-fixed paraffin embedded (FFPE) tissue samples. The baseline characteristics, including age, gender, TNM stage according to the American Joint Committee on Cancer (AJCC) staging 7th edition, and tumor histologic differentiation were collected from the patients’ medical records and surgical pathologic reports. This study was approved by the institutional review board at KNUMC and informed consent was obtained from all the patients included in this study.

### miRNA expression analyses

The total RNA (including miRNA) was extracted from Formalin-fixed, paraffin-embedded (FFPE) specimens using a Recover All^™^ Total Nucleic Acid Isolation Kit (Ambion by Life Technologies^™^, Carlsbad, CA) according to the manufacturer's instructions. The quantity and quality of the isolated total RNA were measured using a NanoDrop^™^ spectrophotometer. The expression of miR-BART1-5p, miR-BART4-5p, and miR-BART20-5p was analyzed using TaqMan miRNA assays (Applied Biosystems, Foster City, CA), while U6 expression was used as an endogenous control for data normalization. In brief, 10 ng of total RNA was reverse transcribed and complementary DNA used for a real-time RT-PCR, where each PCR reaction was performed in triplicate. The difference between the groups is presented as ΔCt, which indicates the difference between the Ct value for the miRNA of interest and the Ct value for the normalizer miRNA. The real-time PCR analyses were performed using a QuantStudio 6 Flex Real-Time PCR System (Applied Biosystems). The median expression level of each miR-BART miRNA was used as the cutoff point, and all cases were subdivided into BART miRNA-high and BART miRNA-low based on the cutoff point.

### Statistical analyses

The descriptive statistics are reported as proportions and medians. The categorical variables were evaluated using a χ2 test, Fisher's exact test, or McNemar test, as appropriate. RFS was defined as the time between diagnosis to tumor recurrence or death from any cause. OS was calculated from the date of diagnosis to death from any cause. Data were censored if patients were free of recurrence or alive at the last follow-up. The Kaplan-Meier method was used to estimate the RFS and OS. The survival curves were compared using a log rank test according to the miRNA expression differences. Multivariate survival analyses were carried out using the Cox proportional hazard regression model. A *p* value < 0.05 was considered statistically significant. The statistical analyses were performed using SPSS for Windows (version 19.0, SPSS Inc., Chicago, Ill., USA).

## References

[1] Bass AJ, Thorsson V, Shmulevich I, Reynolds SM, Miller M, Bernard B, Hinoue T, Laird PW, Curtis C, Shen H, Weisenberger DJ, Schultz N, Shen R (2014). Comprehensive molecular characterization of gastric adenocarcinoma. Nature.

[2] Fuentes-Pananá EM, Morales-Sánchez A (2016). Epstein-Barr Virus-associated Gastric Cancer and Potential Mechanisms of Oncogenesis. Curr Cancer Drug Targets.

[3] Yau TO, Tang CM, Yu J (2014). Epigenetic dysregulation in Epstein-Barr virus-associated gastric carcinoma: Disease and treatments. World J Gastroenterol.

[4] Giudice A, D’Arena G, Crispo A, Tecce MF, Nocerino F, Grimaldi M, Rotondo E, D’Ursi AM, Scrima M, Galdiero M, Ciliberto G, Capunzo M, Franci G (2016). Role of Viral miRNAs and Epigenetic Modifications in Epstein-Barr Virus-Associated Gastric Carcinogenesis. Oxid Med Cell Longev.

[5] Bartel DP (2004). MicroRNAs: Genomics, Biogenesis, Mechanism, and Function. Cell.

[6] Bartel DP (2009). MicroRNA Target Recognition and Regulatory Functions. Cell.

[7] Cai X, Schäfer A, Lu S, Bilello JP, Desrosiers RC, Edwards R, Raab-Traub N, Cullen BR (2006). Epstein–Barr Virus MicroRNAs Are Evolutionarily Conserved and Differentially Expressed. PLoS Pathog.

[8] Lo AK, To KF, Lo KW, Lung RW, Hui JW, Liao G, Hayward SD (2007). Modulation of LMP1 protein expression by EBV-encoded microRNAs. Proc Natl Acad Sci USA.

[9] Lung RW, Tong JH, Sung YM, Leung PS, Ng DC, Chau SL, Chan AW, Ng EK, Lo KW, To KF Modulation of LMP2A expression by a newly identified Epstein-Barr virus-encoded microRNA miR-BART22. Neoplasia.

[10] Riley KJ, Rabinowitz GS, Yario TA, Luna JM, Darnell RB, Steitz JA (2012). EBV and human microRNAs co-target oncogenic and apoptotic viral and human genes during latency. The EMBO J.

[11] Skalsky RL, Corcoran DL, Gottwein E, Frank CL, Kang D, Hafner M, Nusbaum JD, Feederle R, Delecluse H-J, Luftig MA, Tuschl T, Ohler U, Cullen BR (2012). The Viral and Cellular MicroRNA Targetome in Lymphoblastoid Cell Lines. PLoS Pathog.

[12] Shinozaki-Ushiku A, Kunita A, Isogai M, Hibiya T, Ushiku T, Takada K, Fukayama M (2015). Profiling of Virus-Encoded MicroRNAs in Epstein-Barr Virus-Associated Gastric Carcinoma and Their Roles in Gastric Carcinogenesis. J Virol.

[13] Kim H, Choi H, Lee SK (2015). Epstein–Barr virus miR-BART20-5p regulates cell proliferation and apoptosis by targeting BAD. Cancer Lett.

[14] Kim H, Choi H, Lee SK (2016). Epstein-Barr Virus MicroRNA miR-BART20-5p Suppresses Lytic Induction by Inhibiting BAD-Mediated caspase-3-Dependent Apoptosis. J Virol.

[15] Kanda T, Miyata M, Kano M, Kondo S, Yoshizaki T, Iizasa H (2015). Clustered MicroRNAs of the Epstein-Barr Virus Cooperatively Downregulate an Epithelial Cell-Specific Metastasis Suppressor. J Virol.

[16] Edwards RH, Marquitz AR, Raab-Traub N (2008). Epstein-Barr Virus BART MicroRNAs Are Produced from a Large Intron prior to Splicing. J Virol.

[17] Choi H, Lee H, Kim SR, Gho YS, Lee SK (2013). Epstein-Barr Virus-Encoded MicroRNA BART15-3p Promotes Cell Apoptosis Partially by Targeting BRUCE. J Virol.

[18] Choy EY, Siu KL, Kok KH, Lung RW, Tsang CM, To KF, Kwong DL, Tsao SW, Jin DY (2008). An Epstein-Barr virus-encoded microRNA targets PUMA to promote host cell survival. J Exp Med.

[19] Dölken L, Malterer G, Erhard F, Kothe S, Friedel CC, Suffert G, Marcinowski L, Motsch N, Barth S, Beitzinger M, Lieber D, Bailer SM, Hoffmann R (2010). Systematic Analysis of Viral and Cellular MicroRNA Targets in Cells Latently Infected with Human γ-Herpesviruses by RISC Immunoprecipitation Assay. Cell Host Microbe.

[20] Jung YJ, Choi H, Kim H, Lee SK (2014). MicroRNA miR-BART20-5p Stabilizes Epstein-Barr Virus Latency by Directly Targeting BZLF1 and BRLF1. J Virol.

[21] Kang BW, Seo AN, Yoon S, Bae HI, Jeon SW, Kwon OK, Chung HY, Yu W, Kang H, Kim JG (2016). Prognostic value of tumor-infiltrating lymphocytes in Epstein-Barr virus-associated gastric cancer. Ann Oncol.

